# Resveratrol suppresses human colon cancer cell proliferation and induces apoptosis via targeting the pentose phosphate and the talin-FAK signaling pathways-A proteomic approach

**DOI:** 10.1186/1477-5956-9-49

**Published:** 2011-08-17

**Authors:** Jairam Vanamala, Sridhar Radhakrishnan, Lavanya Reddivari, Vadiraja B Bhat, Andrey Ptitsyn

**Affiliations:** 1 Department of Food Science and Human Nutrition, Colorado State University, Fort Collins, Colorado, USA; 2 Cancer Prevention and Control Program, University of Colorado Cancer Center, Aurora, Colorado, USA; 3 Department of Pathology, Scott & White Hospital, Temple, Texas, USA; 4 Department of Microbiology, Immunology and Pathology, Colorado State University, Fort Collins, Colorado, USA; 5 Agilent Technologies, Wilmington, Delaware, USA

**Keywords:** Resveratrol, Proteomics, Talin, Focal Adhesion Kinase (FAK), Pentose Phosphate Pathway, Insulin-like Growth Factor-1 (IGF-1)

## Abstract

**Background:**

We and others have previously reported that resveratrol (RSV) suppresses colon cancer cell proliferation and elevates apoptosis *in vitro *and/or *in vivo*, however molecular mechanisms are not fully elucidated. Particularly, little information is available on RSV's effects on metabolic pathways and the cell-extra cellular matrix (ECM) communication that are critical for cancer cell growth. To identify important targets of RSV, we analyzed whole protein fractions from HT-29 advanced human colon cancer cell line treated with solvent control, IGF-1 (10 nM) and RSV (150 μM) using LC/MS/MS-Mud PIT (Multidimensional Protein Identification Technology).

**Results:**

Pentose phosphate pathway (PPP), a vital metabolic pathway for cell cycle progression, was elevated and suppressed by IGF-1 and RSV, respectively in the HT-29 cell line. Enzymatic assays confirmed RSV suppression of glucose-6 phosphate dehydrogenase (rate limiting) and transketolase, key enzymes of the PPP. RSV (150 μM) suppressed, whereas IGF-1 (10 nM) elevated focal adhesion complex (FAC) proteins, talin and pFAK, critical for the cell-ECM communication. Western blotting analyses confirmed the suppression or elevation of these proteins in HT-29 cancer cells treated with RSV or IGF-1, respectively.

**Conclusions:**

Proteomic analysis enabled us to establish PPP and the talin-pFAK as targets of RSV which suppress cancer cell proliferation and induce apoptosis in the colon cancer cell line HT-29. RSV (150 μM) suppressed these pathways in the presence and absence of IGF-1, suggesting its role as a chemo-preventive agent even in obese condition.

## Background

Cancer is a multifactorial disease whereas cancer cells can upregulate multiple defense mechanisms to evade drug treatments and therapies. It is therefore important to study the different mechanisms of compounds showing promise in chemopreventive efficacy to further enhance targeted therapy development. Proteomic analysis, a powerful method for discovery of new biomarkers and pathways, has recently been used in studies of obesity, diabetes, and especially cancer [1-3]. Proteomic profiling not only offers a method to study cancer but has also indeed broadened our understanding of multiple cancers. Nowadays, profiling and discovery of novel biomarkers are required for not only diagnosis but also for accurate understanding of mechanisms and causes of metabolic disorders [2].

Resveratrol (RSV, 3,5,4'-trihydroxy-trans-stilbene), a stilbenoid and a potent chemopreventive bioactive compound, is found in the skin of red grapes, and peanuts. RSV exerts anti-cancer properties by inhibiting three major stages of carcinogenesis, namely tumor initiation, promotion and progression [4]. RSV has been studied extensively as a chemopreventive/anti-proliferative agent in multiple cancer types including colon and prostate cancers [5,6]. We reported previously that RSV suppressed colon cancer cell proliferation and induced apoptosis even in the presence of IGF-1, a well-known growth factor elevated during obesity which has shown to enrich colon cancer stem cell populations [6,7]. RSV targets p53 and IGF-1R/Wnt signaling pathways to suppress colon cancer cell proliferation and induce apoptosis. RSV interactions with p53, Akt and other effector proteins that regulate proliferation and apoptosis are well documented [6,8-13]. However, the effects of RSV on metabolic pathways like the pentose phosphate pathway (PPP) and the cell-extracellular matrix (ECM) protein interaction, important in cancer cell growth and proliferation are not clearly understood.

High concentrations of RSV used in this study and other studies on colon cancer cell lines are quite achievable in the colon (luminal- as epithelial cells are exposed to RSV directly) with novel pectin based formulations, as most of the trans-resveratrol reaches the colon unaltered. The pectin formulation protects RSV from upper GI tract enzymes and allows for targeted release in the colon (i.e., colon-specific drug delivery system). Pectin (biopolymer) used in such formulations is safe for oral intake and almost completely degraded by colonic bacteria [14-16]. Moreover, studies with human subjects revealed that resveratrol is well tolerated even at high oral doses (daily intake of up to 5 g) without adverse effects [17-19]. Juan *et al *[20] suggested that wines with 14.3 mg/L RSV could provide a luminal concentration of around 80 μM of trans resveratrol. Thus, novel formulations that protect RSV from first-pass metabolism or presystemic metabolism during GI transit and with RSV being safely tolerated in high doses, it is quite possible to achieve 100-150 μM RSV in the colon. Thus, use of dietary bioactive compounds at pharmacological doses, is emerging as a therapeutic approach to target colon cancer in humans.

Over-activation of the IGF system is frequently observed in obese conditions and plays a key role in obesity-promoted colon cancer [21]. Activation of the IGF system due to elevated circulating levels of IGF-1 stimulates colonocyte proliferation [21-24]. IGF-1 bound to IGF-1R activates downstream signaling pathways to promote proliferation and cell cycle progression [25,26]. We have previously shown that RSV suppressed IGF-1 signaling via suppression of IGF-1R resulting in G1 cell cycle arrest and suppression of proliferation in HT-29 and SW480 colon cancer cells [6].

The metabolic pentose phosphate pathway (PPP) produces ribose-5-phosphate required for the synthesis of nucleotides. Cell proliferation requires metabolic sources for the duplication of DNA and cell size [27]. Therefore, PPP is frequently up-regulated in cancer cells; especially in HT-29 cells where, it has been shown that the PPP is very important for cell cycle progression [28]. This pathway is divided into two branches. The oxidative branch uses glucose-6-phosphate as a substrate to generate NADPH, NADPH acts as a reducing agent for the maintenance of reduced glutathione levels and fatty acid synthesis. On the other hand, the non-oxidative branch recycles pentose phosphates to glycolytic intermediates and generates *de novo *ribose-5-phosphate for nucleotide biosynthesis. Key regulatory enzymes of PPP are glucose-6-phosphate dehydrogenase (G6PDH) rate limiting enzyme in the oxidative branch and transketolase (TKT) in the non-oxidative branch [28,29].

Cell-cell and cell-ECM interactions are important for viability and cell growth. Cell adhesion and migration contribute to normal processes such as differentiation, embryonic development, and wound healing. However, in cancer cells such processes are also significant for invasion and metastasis [30]. Key mechanistic steps in these processes involve the extracellular protein interaction with cell specific adhesive receptors such as integrins [31]. In cancer cells, these interactions serve as a link between extracellular and intracellular signals and regulate cell adhesion leading to invasion, proliferation, anoikis (anchorage independent apoptosis), survival and tumor progression [32]. Even though interruption of cell-ECM adhesion is a potential strategy for cancer prevention and treatment, and in spite of the cell-ECM components frequently modulated in chemoprevention studies, not much is known about the potential effects of the bioactive compounds like RSV on cell-ECM and integrin dynamics [33]. Talin is an integrin regulatory protein that translates the external message into regulatory intracellular signal transduction cascades. Cell-ECM interactions mediated via integrin/talin convey vital information to the cell interior to regulate cell proliferation and differentiation [34]. Focal adhesion kinase (FAK) is another member in the family of molecules that regulates cell adhesion dynamics, stimulates multiple cellular signal transduction events leading to cell motility, proliferation and survival [35,36]. Talin has also been suggested to mediate FAK activation upon integrin stimulation [34]. FAK and IGF-1R have been shown to activate common pathways, leading to increased cell proliferation and survival. These studies suggest that talin-FAK signaling plays an important role in signaling initiated by integrins that translates into downstream signaling pathways [34].

In the present study, we used the power of functional proteomics to unravel critical components of the cancer chemoprevention ability of RSV in HT-29 advanced human colon cancer cells. The whole protein fraction of RSV (150 μM) or IGF-1 (10 nM) treated HT-29 cells was analyzed using LC/MS/MS. IGF-1 elevated and RSV suppressed G6PDH and TKT, the 2 key enzymes of oxidative and non-oxidative branches of the PPP, respectively, indicating that RSV suppressed cell cycle progression of HT-29 cells by down-regulating the PPP. Proteins in the focal adhesion complex were also found to be differentially regulated by RSV and IGF-1. RSV (150 μM) suppressed the talin and phosphorylated Fak protein levels even in the presence of IGF-1, a potent mitogen, indicating that RSV anti-cancer effects against human colon cancer cell might be partly due to disruption of cell-ECM interaction. On the basis of these results, we found the PPP and the talin-FAK signaling as critical targets of RSV.

## Results

### Proteomic Analysis of HT-29 colon cancer cells treated with RSV and IGF-1

**Figure 1:**
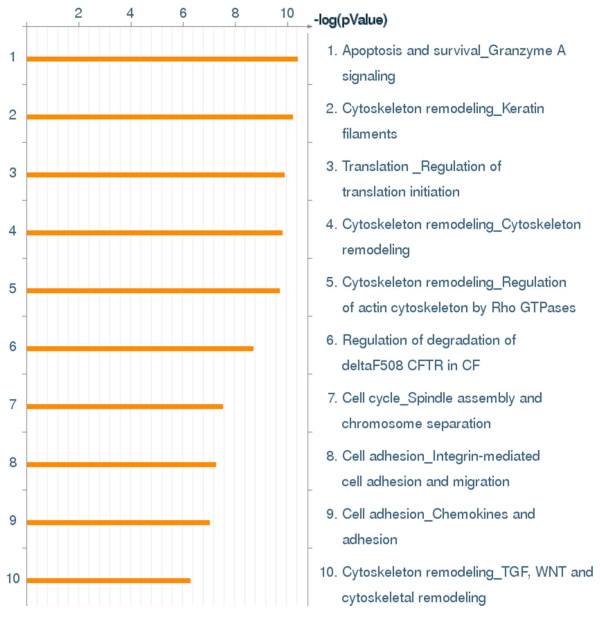
**First 10 most differentially represented biological pathways identified in the set of proteins across all experimental conditions**. The complete list of top 50 statistically significant pathways is given in Additional file 2, Figure S2.

HT-29 cells were treated with either DMSO (solvent control), IGF-1 (10 nM) or RSV (150 μM). LC/MS/MS-Mud PIT analysis of HT-29 cells detected 1231 proteins among all three treatment groups. Using quantile normalization technique allowed us to scale all protein abundances in all samples to the same distribution. This allows realistic estimation of fold change between experimental conditions (Additional File 1, Figure S1). The list of identified proteins and respective normalized fold changes were imported into GeneGo Metacore software to analyze cellular processes significantly altered by IGF-1 and RSV with respect to control. Fifty most differentially represented biological pathways were identified in the set of proteins across all experimental conditions and are presented in Figure S2 (Additional file 2, Figure S2). Major pathways affected by RSV and IGF-1 include those responsible for cytoskeleton remodeling, apoptosis signaling, cell cycle, cell adhesion, inflammation and glucose metabolism (top 10 pathways presented in Figure 1). Pathways that had p < 0.05 were considered significant. RSV responsive pathways, the PPP and the cytoskeleton remodeling pathway, which play a critical role in cancer cell kinetics, were chosen to determine if RSV suppression of proliferation and induction of apoptosis involves suppression of metabolic pathways such as the PPP and disruption of cell-ECM crosstalk.

### IGF-1 and RSV modulate the PPP to target cell cycle progression in human colon cancer cell line HT29

**Figure 2:**
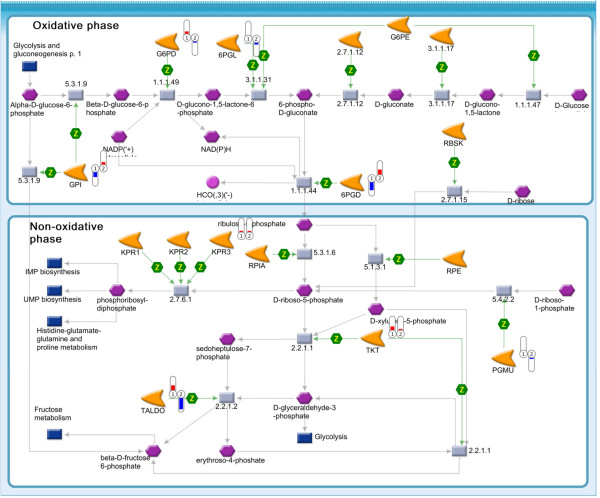
**Map of pentose phosphate pathway (Genego Metacore) overlapped with quantile-normalized estimations of protein abundance**. Flags indicate direction of change (red, up-increased, blue; down-decreased) and fold change (length) of protein abundance in relation to solvent control. 1, IGF-1 and 2, Resveratrol. Results were expressed as mean for three replicate experiments for each treatment group.

**Figure 3:**
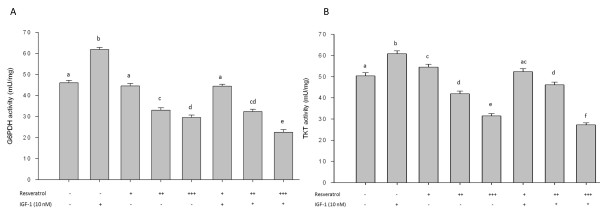
**Effect of RSV (50, 100 and 150 μM denoted as +, ++ and +++ respectively) and/or IGF-1 (10 nM) on G6PDH (A) and TKT (B) enzyme activities in HT-29 colon cancer cells**. HT-29 cells were treated with RSV and/or IGF-1 for 24 h and G6PDH and TKT enzyme activities were analyzed as described in materials and methods. Results were expressed as means ± SE for three replicate experiments for each treatment group. Means that differ by a common letter (a, b, c, d, e, f) differ (p < 0.05).

PPP is frequently up-regulated in cancer cells. Proteomic analysis revealed that G6PDH and TKT the two key enzymes of the oxidative and non-oxidative branches of the PPP respectively, were altered by RSV or IGF-1 treatment. We observed that RSV attenuated G6PDH and TKT levels (Figure 2). Results using enzyme kinetic assays for both G6PDH and TKT support these observations. RSV (100-150 μM) suppressed and IGF-1 elevated both G6PDH and TKT levels. Even in the presence of IGF-1, RSV suppressed G6PDH and TKT enzymatic levels confirming its efficacy in obese conditions (Figure 3A and 3B). Potentiated G6PDH and TKT suppression by RSV (150 μM) in the presence of IGF-1 may be due to up-regulation of reactive oxygen species by both IGF-1 and RSV at high concentration leading to elevated apoptosis [6,37-39].

### RSV suppresses while IGF-1 elevates the talin-Fak signaling pathway

**Figure 4:**
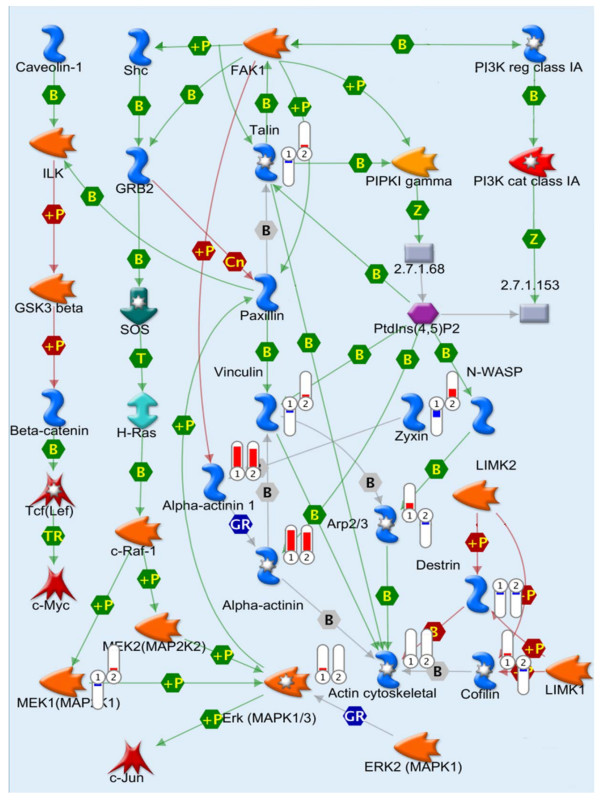
**Map of cytoskeleton remodeling pathway (Genego Metacore, fragment) overlapped with quantile-normalized estimations of protein abundance**. Flags indicate direction of change (red, up-increased, blue; down-decreased) and fold change (length) of protein abundance in relation to non-treated control. 1, IGF-1 and 2, Resveratrol. Results were expressed as mean for three replicate experiments for each treatment group.

**Figure 5:**
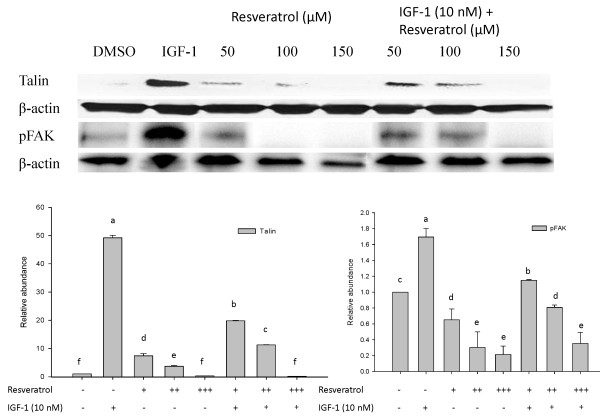
**Effect of RSV (50, 100 and 150 μM denoted as +, ++ and +++ respectively) and/or IGF-1 (10 nM) on talin and pFAK proteins in HT-29 colon cancer cells**. Western blot analysis was performed as described in materials and methods. β-actin served as a loading control. Results were expressed as mean ± SE for three replicate experiments for each treatment group. Means that differ by a common letter (a, b, c, d, e, f) differ (p < 0.05).

Proteomic analysis revealed that the cytoskeleton remodeling pathway is one of the critical pathways (4^th^) altered in the HT-29 colon cancer cells treated with RSV or IGF-1 (Figure 1). As cytoskeletal components make up a major portion of total protein in most tissues, it is not surprising to see quantitative changes in this category of proteins. One of the major signaling pathways in the cytoskeleton remodeling, is the interaction between proteins talin and FAK. Both RSV and IGF-1 altered the talin-FAK signaling pathway, but the differences were not significant (Figure 4). However, activation of talin-FAK signaling and up-regulation of phosphorylated FAK levels have been observed in variety of cancer cells [34,36,40-43]. The levels of talin and pFAK were measured using western blots. Confirmatory results using western blot demonstrated that IGF-1 elevated and RSV (150 μM) suppressed talin and phosphorylated (activated) FAK levels (Figure 5). At lower concentrations RSV increased the talin expression but at higher concentration suppressed the talin expression. This may be due to differential activity of RSV at low vs high concentration-anti-oxidant at low concentration and pro-oxidant at high [38,44]. At all concentrations RSV suppressed IGF-1 induced talin and pFAK levels (Figure 5) confirming efficacy of RSV in the presence of IGF-1.

## Discussion

The modern-day approach to cancer management is multidisciplinary, consisting of surgery, radiation therapy and chemotherapy with potential side effects. Several investigations are underway to improve the efficacy of these treatment modalities or to find new ways to treat or prevent cancer. Proteomics technology plays an important role in finding and validating biomarkers for cancer. Bioactive compounds like RSV have multiple mechanisms of action. It is vital to discover novel targets/biomarkers of chemopreventive agents like RSV that has multiple mechanisms of action so that those targets could be harnessed to develop targeted therapies. The goal of the study was to identify RSV target proteins and mechanisms implicated in its anti-neoplastic activities. The use of the proteomics technology enabled us to identify an array of proteins modulated by RSV. Although our present analysis accessed a small window in the cellular proteome, it was possible to detect RSV modulation of 1231 proteins in the HT-29 cell line. To determine if the expression of many of these proteomic markers was modified at both low and high concentrations of RSV, our confirmatory analysis included western blots and enzyme kinetic assays using RSV at concentrations 50-150 μM alone or after pre incubation with IGF-1, a mitogen elevated during obesity.

We have previously reported that at concentrations > 100 μM RSV suppressed colon cancer proliferation and up-regulated apoptosis even in the presence of IGF-1, elevated during obesity, and that has shown to enrich colon cancer stem cell populations [6,7]. Apoptotic levels correlated with pp53 levels and suppression of proliferation transpired via attenuation of IGF-1R/Akt/Wnt signaling pathways. The present study focuses on novel targets of RSV that govern the proliferation, apoptosis and growth of a cancer cell. Using the pathway analysis software the important targets from the pool of proteins differentially altered by IGF-1 or RSV treatment were selected to devise pathways that have not been studied.

Genego Metacore software provided a basis to evaluate pathways based on differentially expressed proteins. As expected major canonical pathways affected were from the actin cytoskeleton signaling, oxidative stress response pathways, glucose metabolism, apoptosis and cell cycle etc. Among the significantly expressed proteins, G6PDH and TKT from the pentose phosphate pathway family and talin and FAK from the cytoskeleton family were found to be differentially expressed in RSV or IGF-1 treatments (Figure 1, 2 and 4). This along with the data showing that the glycolytic pathways [45,46] and the cell-ECM interaction [1] to be frequently deregulated in cancer cells, fueled interest in RSV, the PPP and the talin-pFAK interaction.

Cell division is an energy-demanding process and its correct progression depends on sufficient metabolic resources to support a doubling of cell mass. Though nutrient availability is a key factor for cell proliferation, nucleotide synthesis is a rate limiting step in cancer cell replication [29]. Ribose-5-phosphate, which is a key nucleotide component, is synthesized from glycolytic intermediates in the PPP. PPP is considered important in tumor proliferation processes because of its role in supplying tumor cells with reduced NADP and carbons for intracellular anabolic processes [27]. In particular, the two key enzymes G6PDH and TKT have been shown to play a critical role in cancer cell cycle progression in the HT-29 cell line [28,29].

In this study, we have demonstrated, using two different experimental approaches, that the PPP which is specifically elevated during cell cycle progression in the highly proliferating advanced human adenocarcinoma cell line HT-29 [28,29] is further elevated by IGF-1 but suppressed by the bioactive compound RSV. Thus, a specific decrease in the activity of 2 key enzymes G6PDH and TKT, may lead to suppression of PPP that provides precursors of nucleotides for cancer cell cycle progression. Resveratrol at high concentration (150 μM) showed pronounced suppression of G6PDH and TKT in the presence of IGF-1. IGF-1 has been shown to elevate cancer cell proliferation via elevation of ROS (reactive oxygen species) [39]. At high concentrations where resveratrol has been shown to be pro-oxidant, the pronounced effect of resveratrol on pentose phosphate pathway in the presence of IGF-1 may be due to elevated ROS levels that promotes apoptosis [20,44]. Detailed mechanistic reason for this potentiated effect is still need to be delineated, however, based on our earlier studies we propose that this might be due to elevated suppression of pAKT, Cyclin D1, nuclear β-catenin and SP1, proteins critical for cancer cell proliferation and cell cycle progression, in the presence of IGF-1 by resveratrol [6].

Suppression of PPP by RSV may be via inhibition of mTORC1. Recent evidence has shown clearly that the mTORC1 controls downstream metabolic pathways like the PPP via S6K1/SREBP-1/2 [47]. Our earlier experiments with mTOR signaling revealed that RSV activated tuberous sclerosis protein 2 (TSC-2, an mTORC1 inhibitor) and suppressed two best characterized downstream targets of mTORC1 (mammalian target of rapamycin complex 1), p70S6 kinase 1 (S6K1) and eukaryotic initiation factor 4E (eIF4E) binding protein 1 (4E-BP1) [48].

Talin-FAK interaction has been well established and is implicated in various cancers [49,50]. Talin plays an essential role in integrin activation and acts as a link between cell and ECM to regulate cancer cell kinetics [49]. Talin has been shown to engage in focal adhesion interactions with Akt signaling as the intracellular survival mechanism to confer anoikis (anchorage independent apoptosis) [51,52] resistance and promote cancer cell invasion, especially in prostate cancer [49]. IGF-1 has been consistently linked to increased cell proliferation and cell migration, elevating cell invasion and metastatic properties of cancer cells [53]. Our earlier work with systems biology to identify biomarkers in metastatic progression of cancer featured talin as one of the differentially expressed genes in metastatic tumors in the context of cytoskeleton remodeling pathway [1]. Even the current proteomics data had talin as one of the differentially expressed proteins in IGF-1 and RSV treatments (Figure 4). RSV elevated talin levels at low concentration (50 μM) and suppressed talin, and concurrently elevated apoptosis at high concentration (150 μM). This may be due to the action of RSV as an antioxidant at low concentrations and pro-oxidant at high concentrations [38,44,54]. Anti-oxidant action at lower doses could protect DNA damage via scavenging of ROS, whereas at high concentration RSV acts as pro-oxidant leading to oxidative breakage of cellular DNA in the presence of transition metal ions such as copper causing apoptosis [44,54]. This could possibly explain differences in talin activity at low vs high concentrations of RSV (RSV at 50 μM is not sufficient to induce apoptosis [20]). However, RSV was effective in suppressing IGF-1 stimulated talin expression, irrespective of concentration used. "FAK carries out protein-protein interaction adaptor functions at sites of cell attachment to the extracellular matrix (ECM), thereby contributing to focal-adhesion 'scaffolding'. FAK also transmits adhesion-dependent and growth-factor-dependent signals into the cell interior [50,55]." The synergistic signaling between growth-factor receptors like IGF-1R and FAK might be particularly relevant as both are often up-regulated in tumor cells [36,55]. FAK has also been shown to work similar to the IGF-1R to activate common pathways, leading to increased proliferation and cell survival. At least in pancreatic cancer, it has been shown that dual inhibition of FAK and IGF-1R led to a synergistic decrease in cell proliferation and increase in cell detachment and apoptosis compared with inhibition of either pathway alone [56,57]. We have shown previously that RSV inhibits IGF-1R in HT-29 cells [6]. RSV suppressed FAK activation in the presence and absence of IGF-1. These results indicate that RSV suppression of cell proliferation and elevation of apoptosis involves modulation of FAK signaling, considering that the integrin-mediated FAK signaling regulates both proliferative and apoptotic signaling pathways [58,59].

## Conclusions

Proteomic profiling enabled us to identify novel targets of RSV. Our results establish PPP and the talin-pFAK as targets of RSV to suppress cancer cell proliferation and induce apoptosis in colon cancer cell line HT-29. These studies may prove germane to the envisaged use of RSV as a colon cancer chemopreventive agent as well as provide novel biomarkers to target and halt colon cancer cell kinetics.

## Materials and methods

### Chemicals

RSV and other cell culture materials were obtained from Sigma Chemical Co. (St. Louis, MO). IGF-1 was purchased from R&D Systems (Minneapolis, MN). Fetal bovine serum (FBS) was obtained from HyClone (Thermo Fisher Scientific, Inc., Pittsburgh, PA).

### Cell line

Colon cancer cell line HT-29, was obtained from the American Type Culture Collection (Manassas, VA). Cells were maintained at 37°C in a humidified atmosphere with 5% CO_2 _and grown in Dulbecco's Modified Eagle's Medium (DMEM) F-12 supplemented with 10% fetal bovine serum (FBS), 2.2 g/L sodium bicarbonate, 0.2 g/L bovine serum albumin and 10 mL/L streptomycin-penicillin mix.

### Sample preparation

HT-29 cells were seeded at a density of 1.5 × 10^5 ^cells/mL in DMEM F-12 media with 5% charcoal-stripped FBS. Next day, cells were treated with DMSO (solvent control), IGF-1 (10 nM) or RSV (150 μM) for 24 h. We found from dose response studies with IGF-1 (5-20 nM) that 10 and 20 nM IGF-1 treatments did not differ (p < 0.05) in elevating cell proliferation (data not shown). Therefore, we used 10 nM concentration of IGF-1 for our experiments, which is close to normal circulating levels. Protein was extracted into a high-salt buffer containing 1% protease and phosphatase inhibitor cocktail, and protein concentrations were determined by a BCA Protein Assay kit (Pierce, Rockford, IL). The lysate samples (200 μg) were reduced, alkylated and double digested with trypsin to generate peptides. The digested peptides were completely dried in a SpeedVac and suspended in 100 μL of 5% acetonitrile acidified with 0.1% formic acid (mobile phase A). 200 μg of peptides (40 μl) were directly loaded onto a 1 × 150 mm Poly-SEA strong cation exchange column (Michrom Bioresources, Auburn, CA) using Agilent 1200 auto sampler. Peptides were eluted to 10 fractions using 0-100 mM ammonium formate for 40 min (mobile phase B: 1 M ammonium formate, 10% Formic acid in 5% acetonitrile) and 5 fractions in 100-1000 mM ammonium formate for 10 min on Agilent 1200 Capillary LC and Analytical-fraction collector at a flow rate of 50 μL/min. Peptides were dried and reconstituted in 10 μl of 0.1% TFA for LC-MS/MS analysis.

### HPLC-Chip/MS analysis

A 3 μl volume of peptides (30% of SCX fraction) were injected into an LC/MS system consisting of an 1100 Series liquid chromatograph, HPLC-Chip Cube MS interface, and 1100 Series LC/MSD Trap XCT Ultra ion trap mass spectrometer (all Agilent Technologies, Santa Clara, CA). The system was equipped with an HPLC-Chip (Agilent Technologies) that incorporated a 160 nL enrichment column and a 150 mm × 75 μm analytical column packed with Zorbax 300SB-C18 5 μm particles. Peptides were loaded onto the enrichment column with 97% solvent A (water with 0.1% formic acid). They were then eluted with a gradient from 3% to 45% solvent B (acetonitrile with 0.1% formic acid) in 25 min, followed by a steep gradient to 90% solvent B in 5 min at a flow rate of 0.3 μl/min. The total runtime, including column reconditioning, was 35 min. The column effluent was directly coupled to an LC/MSD Trap XCT Ultra ion trap mass spectrometer from Agilent Technologies via a HPLC-Chip Cube nanospray source operated at ~1900 volts in ultra-ultra mode. The gain control (ICC) was set to 500,000 with a maximum accumulation time of 150 milliseconds. Collision induced dissociation (CID) was triggered on the six most abundant, not singly charged peptide ions in the *m/z *range of 450**-**1500. Precursors were set in an exclusion list for 1 min after two MS/MS spectra. Results were expressed as mean for three replicate experiments for each treatment group.

### Data analysis

CID data was searched against the NCBInr human database, using the Agilent Spectrum Mill Server software (Rev A.03.03.) installed on a HP Intel^® ^Xeon (TM) dual processor server. Peak lists were created with the Spectrum Mill Data Extractor program with the following attributes: scans with the same precursor ± 1.4 *m*/*z *were merged within a time frame of ± 15 s. Precursor ions needed to have a minimum signal to noise value of 25. Charges up to a maximum of 7 were assigned to the precursor ion, and the ^12^C peak was determined by the Data Extractor. The NCBInr database was searched for tryptic peptides with a mass tolerance of ± 2.5 Da for the precursor ions and a tolerance of ± 0.7 Da for the fragment ions. Two missed cleavages were allowed. A Spectrum Mill auto validation was performed first in the protein details followed by peptide mode using default values [Minimum scores, minimum scored peak intensity (SPI), forward minus reversed score threshold, and rank 1 minus rank 2 score threshold]. All protein hits found in a distinct database search by Spectrum Mill are non-redundant.

### Data scaling and normalization

Quantitative estimation of differences between physiological states by analysis of proteomics data has a number of challenges. Only a fraction of proteins actually present in each sample is identified and a still smaller fraction is quantified. Technical variation overlapped with these fractions results in substantial differences in the range of variation for identified fraction even though the overall range of variation between samples is similar (see Additional file 1, Figure S1). The resulting distribution of quantified protein abundance units has long tails of low-abundance proteins and proteins identified in only one of the samples. It is commonly advised that for MS proteomics (unlike microarrays) more technical replicates should be done in order to control variation and achieve more reliable quantitative estimation of change between samples. However, this expensive and labor-intensive "brute force" approach is not always feasible. We have taken an alternative data analysis approach which allows quantitative estimation of changes between samples with limited number of estimates. Our method is based on a reasonable assumption of little or no change in abundance for the majority of proteins in all samples. None of the experimental conditions we create in this project can be associated with lethality or high stress. There is also no indication of experiment-induced stress response in the pattern of expressed proteins. Assuming that relatively small fraction of genes have large fold changes we can apply quantile normalization algorithm, similar to that described by Bolstad et al. [60] for microarray normalization: *x_norm _= F^-1^(G(x)), where F is the distribution function of the actual sample, and G is the reference distribution function*. We estimate *G *by the empirical distribution of each sample and *F *by using the empirical distribution of the averaged quantitative estimations of peptide abundance in samples across all experimental conditions, not just replicate groups. This approach has proven to be effective in low-replicate microarray studies [1,61]. In our implementation *G *is more smoothly estimated by application of additional Savitski-Golay polynomial smoothing [62]. In addition after scaling, we zero down scaled values for proteins not identified in particular sample. Quantile normalization scales all protein abundances in all samples to the same distribution and allows realistic estimation of fold change between experimental conditions.

### Biological pathway analysis

All lists of identified proteins and respective normalized expression values were imported to GeneGo Metacore (GeneGo INC, St. Josef, MI). Analysis of overrepresentation of canonical pathways, co-regulation/protein interaction pathways and comparative network analyses are performed using intrinsic tools and pre-formed analysis pipelines of Genego Metacore.

### Western blot analysis

HT-29 cells were seeded at a density of 1.5 × 10^5 ^cells/mL in Dulbecco's Modified Eagle's Medium F-12 with 5% charcoal-stripped fetal bovine serum for 24 h. Cells were treated with solvent control (DMSO), IGF-1 (10 nM), different concentrations of RSV (50, 100 and 150 μM) with and without IGF-1 for 24 h. Protein was extracted into a high-salt buffer containing 1% protease inhibitor cocktail from Sigma-Aldrich (St. Louis, MO), and protein concentrations were determined by a BCA protein assay kit from Pierce (Rockford, IL). Cell lysates (45 μg) were incubated at 98°C for 5 min and separated on 4-12% Criterion XT bis-tris gel at 120 V for 2 h in 1X XT MOPS Running Buffer (Bio-Rad Laboratories, Hercules, CA) and electrophoretically transferred to Immuno-Blot PVDF membranes (Bio-Rad) at 95 V for 35 min in tris-glycine transfer buffer with 0.025% SDS. PVDF membranes were blocked with Superblock buffer (Thermo Fisher Scientific Inc.) for 1 h at room temperature. The membranes were incubated with rabbit polyclonal anti-pFAK antibody (1:500; Cell Signaling, Danvers, MA) or mouse monoclonal anti-talin antibody (1:750; Millipore, Billerica, MA) or goat polyclonal anti-β-actin antibody (1:2500; Santa Cruz Biotechnology, Santa Cruz, CA). Membranes were also probed with respective IgG-HRP secondary antibodies from Santa Cruz Biotechnology (1:20,000; Santa Cruz, CA) and scanned using UVP imaging software (UVP BioDoc-It^® ^Imaging System; Upland, CA). β-actin served as a loading control.

### Enzymatic analysis

G6PDH activity was measured using Glucose-6-Phosphate Dehydrogenase assay kit (Biovision, Mountain View, CA). G6PDH activity was measured at absorbance 450 nm using the manufacturer's protocol. TKT activity was determined using the method of de la Haba et al [63]. Briefly, protein extracts were added to a 96-well plate containing 216 mM glycylglycine, 3.3 mM xylulose-5-phosphate, 1.7 mM ribose 5-phosphate, 0.002% (w/v) cocarboxylase, 0.14 mM ß-nicotinamide adenine dinucleotide (reduced form), 15 mM magnesium chloride, 20 units α-glycerophosphate dehydrogenase/triosephosphate isomerase (based on triosephosphate isomerase units). Decrease in absorbance was recorded over 15 minutes at 340 nm and activity was determined based on the method of de la Haba et al [63]. Protein concentration of cell extracts was determined using the BCA protein assay (Pierce) to calculate the specific activity of the enzymes.

## Conflict of interests

The authors declare that they have no competing interests.

## Authors' contributions

JV, SR and LR performed experiments and drafted the paper. SR ran some of the western blots, enzymatic assays and worked on the first draft of the manuscript with the help of LR and JV. AP helped in statistical analysis and performed pathway analysis using Genego Metacore. VBB helped with MS/MS analysis that was essential to the project. JV conceived the study, participated in its design and coordination, and corrected the manuscript. All authors read and approved the final manuscript.

## Grant Support

This work was supported by College of Applied Human Sciences Challenge Grant (2009-2010) and National Research Initiative Grant 2009-55200-05197 from the USDA National Institute for Food and Agriculture.

## Supplementary Material

Additional file 1 **Box plot of variation between samples before (A) and after (B) quantile normalization**. Click here for file

Additional file 2 **List of all statistically significant differentially represented biological pathways identified in the set of proteins across all experimental conditions**. Click here for file
