# Supplementary figures and images for: Resveratrol suppresses human colon cancer cell proliferation and induces apoptosis via targeting the pentose phosphate and the talin-FAK signaling pathways-A proteomic approach
Source: Proteome Sci. 2011 Aug 17;9:49. doi: 10.1186/1477-5956-9-49 (PMC3175442; doi:10.1186/1477-5956-9-49)

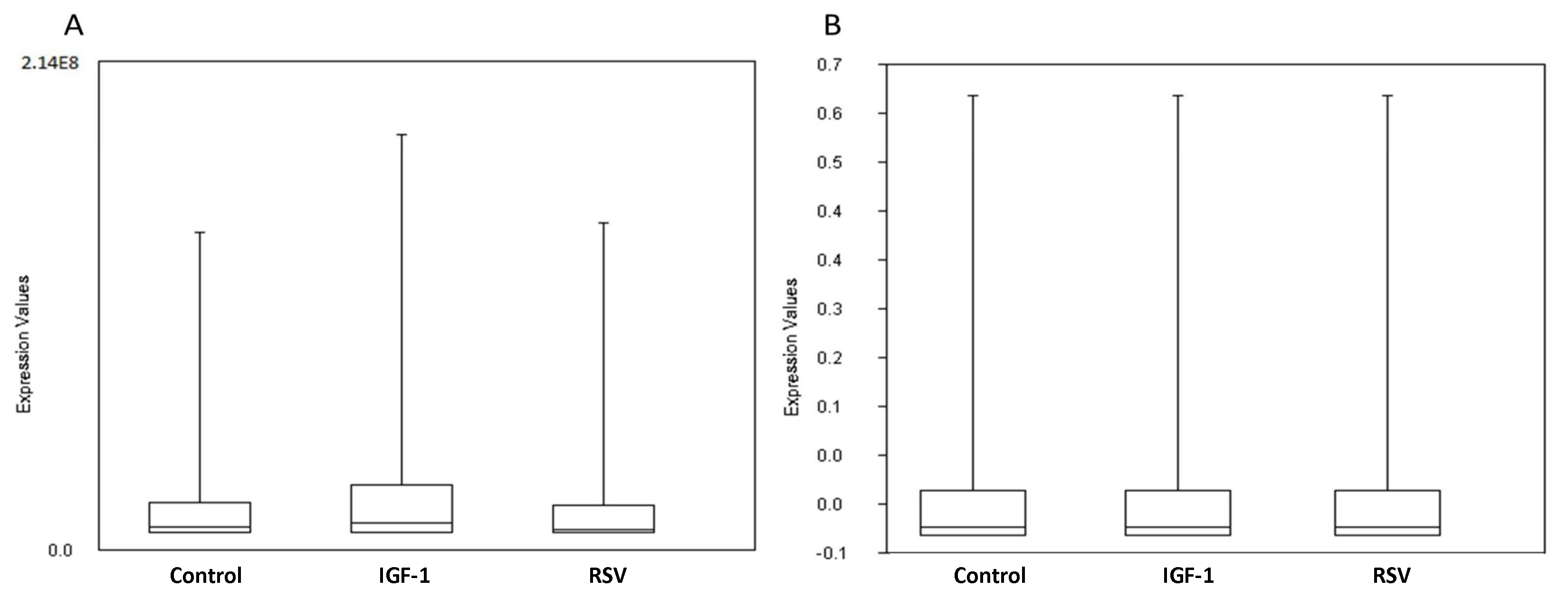

Supplement: Additional file 1 — Box plot of variation between samples before (A) and after (B) quantile normalization. [file 1477-5956-9-49-S1.TIFF]
